# Photoreceptor alteration in intermediate age-related macular degeneration

**DOI:** 10.1038/s41598-020-78201-9

**Published:** 2020-12-03

**Authors:** Enrico Borrelli, Riccardo Sacconi, Biancamaria Zuccaro, Michele Cavalleri, Alessandro Bordato, Ilaria Zucchiatti, Lea Querques, Francesco Bandello, Giuseppe Querques

**Affiliations:** 1 Department of Ophthalmology, Vita-Salute San Raffaele University, Via Olgettina 60, Milan, Italy; 2 IRCCS San Raffaele Scientific Institute, Milan, Italy

**Keywords:** Diagnostic markers, Retinal diseases

## Abstract

The aim of this study was to analyze photoreceptor alterations occurring in eyes with intermediate age-related macular degeneration (AMD) and to investigate their associations with choriocapillaris (CC) flow. In this retrospective case–control study, we collected data from 35 eyes with intermediate AMD from 35 patients who had swept source optical coherence tomography structural and angiography imaging obtained. A control group of 35 eyes from 35 healthy subjects was included for comparison. Our main outcome measure for comparison between groups was the normalized reflectivity of *en face* image segmented at the ellipsoid zone (EZ) level, which was calculated to quantify the photoreceptor damage. OCTA metrics to quantify CC flow signal were also computed. These metrics were measured in a circle centered on the fovea and with a diameter of 5 mm. In intermediate AMD eyes, the macular area occupied by drusen was identified. Therefore, the EZ reflectivity and CC flow signal were separately measured in regions without drusen (“drusen-free” region). Measurements were generated using previously published algorithms. Mean ± SD age was 74.1 ± 6.8 years in the intermediate AMD group and 72.1 ± 6.0 years in the control group (p = 0.206). The normalized EZ reflectivity was 0.76 ± 0.10 in the intermediate AMD group and 0.85 ± 0.08 in the control group (p < .0001). In the “drusen-free” region, the normalized EZ reflectivity was 0.77 ± 0.10 (p < .0001  vs. healthy controls) and was positively correlated with the CC flow signal density (ρ = − 0.340 and p = 0.020). In conclusion, eyes with intermediate AMD exhibit a diffuse reduced EZ normalized reflectivity, and this reduction is correlated with CC flow signal in the regions without drusen. This study supports the concept of the damage of the unit comprised of photoreceptor, CC, and intervening tissues as an early event in AMD.

## Introduction

Age-related macular degeneration (AMD) is a major cause of decreased vision among elderly individuals in developed countries^1^. This disorder may present at different stages and the “intermediate AMD” stage is clinically featured by the presence of pigmentary abnormalities and/or large drusen^2^.

Although the AMD pathogenesis is intricate and related with many systemic and lifestyle factors that may have a role in the development and progression of this disorder^3,4^, a growing evidence suggests that this disorder is ultimately characterized by damage of the unit comprised of photoreceptors, retinal pigment epithelium (RPE), Bruch’s membrane, and choriocapillaris (CC)^5–7^. Importantly, several evidences suggest that this may be considered as a tightly knit, integrated unit^8–10^. In AMD, this impairment causes the development of drusen and progressive photoreceptor, RPE and CC degeneration^11–13^.

Although photoreceptor loss is associated with aging^14^, both histopathological and imaging studies have displayed that intermediate AMD eyes may be characterized by a greater photoreceptor loss^15,16^. Alterations in CC flow are also associated with aging^13,17–19^ and a greater reduction in CC flow was demonstrated to occur in intermediate AMD eyes^20–25^. Therefore, a greater reduction in CC flow in these eyes might provide a potential rationale for a damage in photoreceptors via an ischemic mechanism^11^.

*En face* optical coherence tomography (OCT) imaging provides a topographic qualitative and quantitative assessment of photoreceptors. On commercial structural OCT, the ellipsoid zone (EZ) layer (formerly known as inner segment/outer segment junction) is characterized by an high reflectivity attributed to numerous mitochondria in photoreceptor inner segment ellipsoids^26^. On the *en face* structural OCT images, anomalies in the EZ may be displayed as hyporeflective regions. Therefore, the EZ reflectivity has been employed in several previous reports as a readout of photoreceptor damage^16,27–29^.

In the present study, we provided a quantitative assessment of the photoreceptor damage in intermediate AMD by employing a standardized methodology^16,29^ to objectively measure the EZ reflectivity. Our aim was to explore the relationship between photoreceptor damage and CC flow signal in these eyes. Measurements were performed in a circular region of interest (ROI) centered on the fovea and with a diameter of 5 mm. Furthermore, our analyses were also separately assessed in areas without drusen (“drusen-free” region). The latter choice was consequent to the following reasons: (i) the measurements of the EZ may be altered in regions with drusen because of the occurrence of EZ distortion and/or segmentation errors; (i) the quantification of CC flow signal may be more affected by shadowing artifacts in regions colocalizing with drusen; and (iii) photoreceptors may be regionally affected by drusen through a mechanical compression or by impeding the transition of oxygen and nutrients from the CC to the outer retina. Therefore, the assessment of associations between EZ reflectivity and CC flow signal in regions without drusen was aimed at understanding the relationship between these two variables after restricting the above-mentioned confounding factors. Our findings could be helpful to better comprehend the AMD pathophysiology, and to recognize new targets for therapy.

## Methods

### Study participants

This study was a retrospective case–control analysis. The authors in this study identified patients with a clinical diagnosis of intermediate AMD in at least one eye as determined by clinical examination and by structural OCT^2^. All patients were imaged with the PLEX Elite 9000 device (Carl Zeiss Meditec Inc., Dublin, CA, USA) between July 2018 and October 2019. Written informed consent was obtained from all subjects, and it was approved by the San Raffaele Ethics Committee. The study adhered to the tenets of the Declaration of Helsinki and Health Insurance Portability and Accountability Act.

Exclusion criteria for intermediate AMD eyes were: (i) history of ocular surgery or anti-vascular endothelial growth factor (VEGF) injection; (ii) myopia greater than − 3.00 diopters; (iii) presence of subretinal drusenoid deposits (i.e. reticular pseudodrusen) as determined with OCT analysis^30^, since its presence may alter the assessment of the reflectivity of the *en face* EZ image; (iv) presence of type 1 non-exudative quiescent neovascularization as determined with OCT angiography (OCTA) analysis^31^; and (v) any maculopathy secondary to causes other than AMD.

Because age may influence quantitative measurements of the CC on OCTA^18,32^, a control group similar with respect to age and gender was also included in the current analysis. All control subjects failed to demonstrate evidence of ocular disease or media opacity as evaluated by dilated fundus examination, OCT, and OCTA analysis.

Furthermore, we excluded poor quality images with a signal strength index lower than 7 (a measurement in a scale 0–10 indicating the level of retinal tissue signal with respect to the noise or background level in OCT data), as recommended by manufacturers and applied in previous studies^18,33^.

### Imaging

Patients underwent SS-OCT and SS-OCTA imaging using the PLEX Elite 9000 device (Carl Zeiss Meditec Inc., Dublin, CA, USA) which uses a swept laser source with a central wavelength of 1050 nm (1000–1100 nm full bandwidth) and operating at 100,000 A-scans per second. This instrument employs a full-width at half-maximum (FWHM) axial resolution of approximately 5 μm in tissue, and a lateral resolution at the retinal surface estimated at approximately 14 μm. Structural OCT and OCTA imaging of the macula included a 6 × 6-mm field of view area centered on the fovea (500 A-scans × 500 B-scans).

### Image processing

#### Delineation of the region without drusen

**Figure 1:**
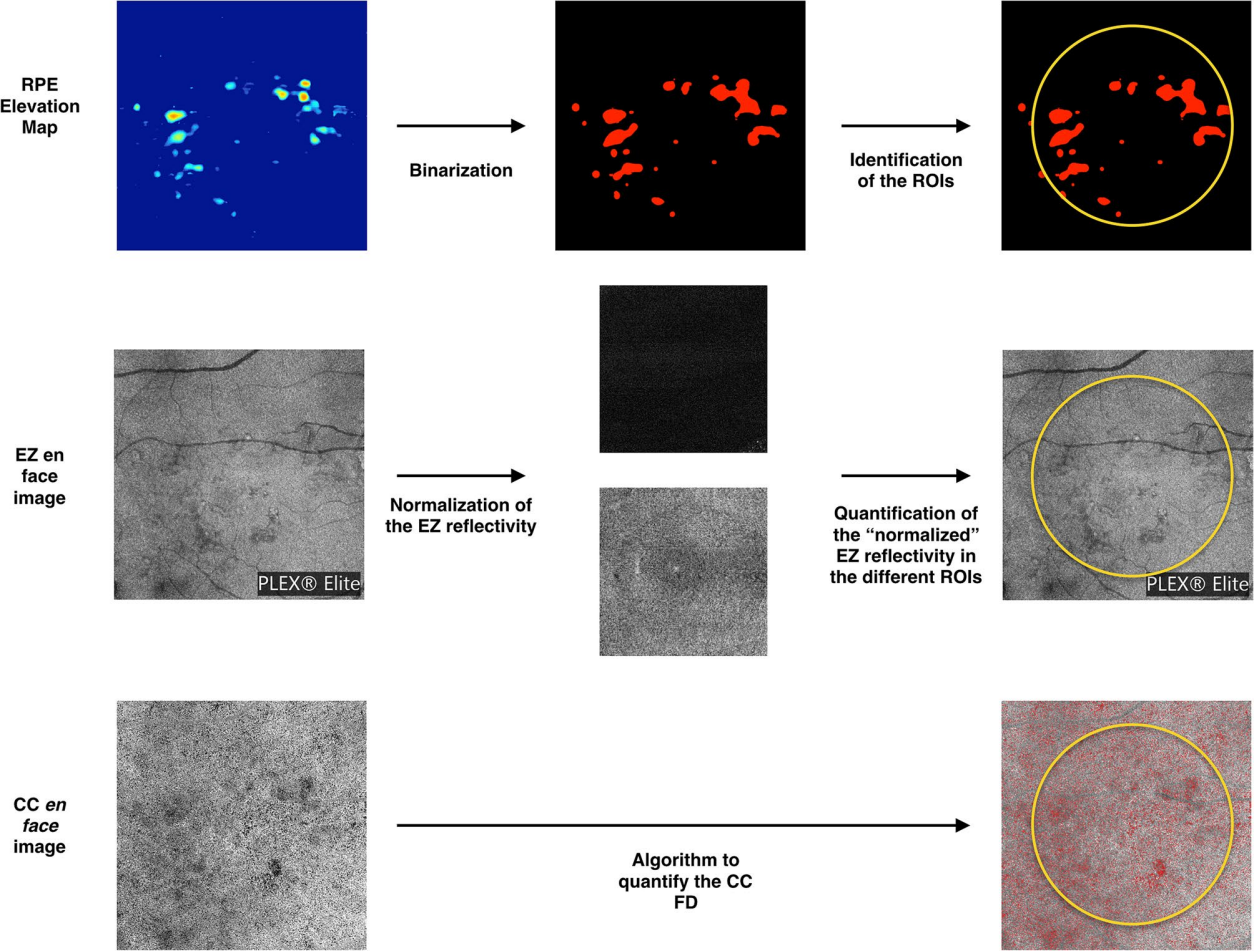
Representation of the algorithm used to investigate the images. The *en face* RPE elevation map image was used to identify regions occupied by drusen. This image was imported into ImageJ, and the “MaxEntropy” threshold was applied to “binarize” this image. In the binarized image, the “drusen” region (red) was excluded in a following analysis which was aimed at measuring metrics in the “drusen-free” region. Measurements were performed in a circular region of interest centered on the fovea (diameter of 5.0 mm—displayed in yellow in the figure). The *en face* image of the ellipsoid zone (EZ) was also imported in ImageJ. A previously described image-processing algorithm using two reference structures, the vitreous and the retinal nerve fiber layer, was employed to calculate the EZ normalized reflectivity. The *en face* flow image of the CC was compensated using the corresponding CC *en face* structural image. A local thresholding method was applied in order to detect pixels falling below this threshold (in red in the lower right image). Values detected in this example are, as follows: drusen area = 1.36 mm^2^; drusen volume = 0.05 mm^3^; normalized EZ reflectivity in the ROI = 0.79; normalized EZ reflectivity in the drusen-free region = 0.80; CC FD% = 24.2%; CC FD% in the drusen-free region = 24.1%.

**Figure 2:**
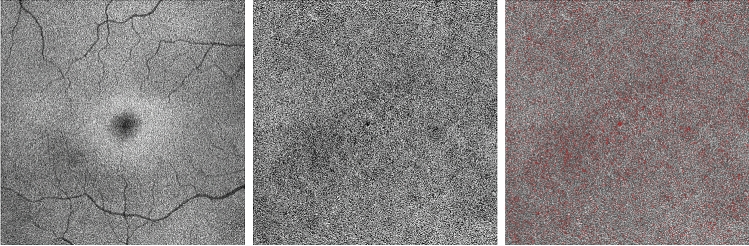
Representative images from an healthy control eye. The *en face* EZ image (middle-left) is obtained with the slab displayed in the reference OCT B-scan image (left). The *en face* EZ image demonstrates a uniform reflectivity which may indicate a normal photoreceptor structure. The original (middle-right) and post-processing (right) CC OCTA images show areas of flow deficits (in red in the right image) which are more localized in the foveal region.

**Figure 3:**
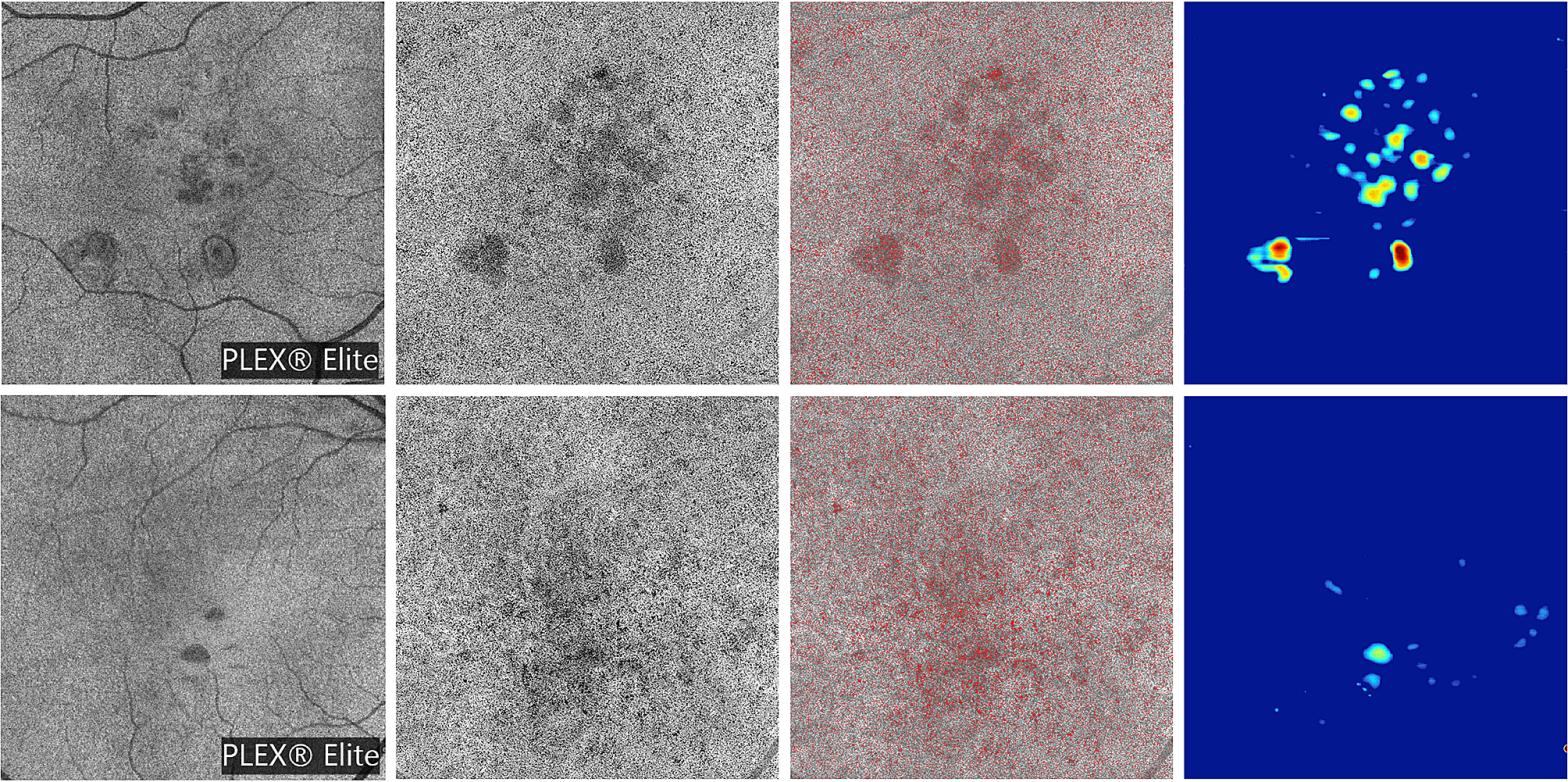
Representative images from intermediate AMD eyes. The OCT B-scan images (first column) show the slab set to visualize the EZ image. The *en face* EZ images (second column) display several regions of reduced reflectivity, this suggesting the presence of areas of photoreceptor impairment that seems to be greater in areas with drusen. The original (third column) and post-processing (fourth column) CC images show areas of increased FD percentage, which are mainly located in the regions occupied by drusen. The *en face* RPE elevation map images (fifth column) display the distribution of drusen in the macular region.

In order to identify the region without drusen (“drusen-free” region), we used the advanced RPE analysis version 0.4 algorithm which is available on the ARI network by Zeiss (https://arinetworkhub.com). This algorithm is aimed at generating the RPE elevation map with respect to Bruch's membrane. The RPE elevation *en face* map is a ".png" image describing the degree of RPE elevation in an *en face* map. In details, different colors indicate the degree of elevation in the form of a heat-map, from 0 µm elevation (in blue) to a maximum of 100 µm elevation (in bright red) (Figs. 1, 2, 3). The algorithm also outputs quantitative data indicating the *en face* area of the RPE that is considerably elevated from Bruch's membrane (drusen area), as well as the volume of such elevation (drusen volume), in a circle centered on the fovea and with a diameter of 5 mm. The position of the fovea was selected manually using the structural OCT. In details, the foveal center was manually annotated on the CC and EZ *en face* images by using the structural OCT volume as reference.

The *en face* elevation map image was imported into ImageJ, and the “MaxEntropy” threshold was applied to “binarize” this image. This binarization threshold was previously used to binarize an elevation map image in intermediate AMD eyes^21^ and also in the present study was evaluated as an effective algorithm to select the drusen region, as evaluated by two experienced graders (EB and RS). In the binarized image, the “drusen” and “drusen-free” regions were displayed with two different colors (Fig. 1).

#### Quantification of the EZ normalized reflectivity

The EZ normalized reflectivity is a dimensionless metric that was measured as previously shown (Fig. 1)^16,29^. In brief, the structural scan of the macula was used to automatically generate the EZ en face image—slab of 21 μm with the inner boundary placed 45 μm above the RPE reference, as previously described^16^. This image was imported into Fiji ImageJ software version 2.0.0 (National Institutes of Health, Bethesda, MD; available at http://rsb.info.nih.gov/ij/index.html). The mean brightness of the EZ *en face* image was thus measured in a circular region of interest centered on the fovea and with a diameter of 5.0 mm. Assuming that the assessment of the EZ may be altered in regions with drusen because of the occurrence of EZ distortion and/or segmentation errors, this measurement was also separately performed in the “drusen-free” regions contained in this circular region of interest. Of note, this measurement was made after excluding the region occupied by major retinal vessels, as previously showed^29^.

Since the brightness of structures displayed using structural OCT may depend on a variety of uncontrollable factors, we employed a previously described image-processing algorithm to normalize the signal across a cohort^16,29,34–36^. For each eye, this algorithm uses the vitreous and the retinal nerve fiber layer (RNFL) as dark and bright references, respectively. After testing the mean brightness in these structures, a formula was applied to obtain the EZ normalized reflectivity for each eye and analyzed region. As the inter-observer agreement for EZ normalized reflectivity algorithm has been reported previously and demonstrated to be excellent in intermediate AMD and control eyes^16^, this was not reassessed in the present study (Figs. 1, 2, 3).

#### Measurement of the CC flow signal

In order to quantify the CC flow signal, we used the multilayer segmentation algorithm (version 0.5) which is available on the ARI network by Zeiss (https://arinetworkhub.com). This algorithm consists on a graph-based approach using a combination of intensity, axial gradient, gradient magnitude and positional information as cost functions to find the optimal location of each layer within a B-scan or several adjacent B-scans. The *en face* OCTA images of the CC were generated using a 20 μm thick slab that followed the contour of the RPE fit boundary, where Bruch’s membrane is usually located, and was placed at about 29 μm under this structure, as set by the manufacturer^37,38^. Importantly, using this algorithm, the RPE fit boundary does not represent a direct segmentation of Bruch’s membrane generated directly from the OCT data, but it is actually a surface fit to the RPE fit boundary excluding any region of pathology.

The obtained image was compensated using the corresponding CC *en face* structural image in order to adjust for shadowing artifacts^22,39,40^ and retinal vessel projection artifacts were therefore removed. The Phansalkar method was used to binarize the images, as previously described^22,32,41–43^. As recently suggested, the Phansalkar threshold was set with a window radius of 3 pixels (~ 103 μm^2^), as recently suggested by Chu and colleagues^44^. In the latter study, the authors proved that the latter radius is the best strategy for analyzing 6 × 6-mm scans with a 1024 × 1024 size. Finally, as suggested by Chu et al.^44^, the isolated regions with an equivalent diameter smaller than 24 μm were subsequently removed from the obtained binarized images, as they were presumed to represent noise^45^. Recent histopathological evidences have questioned this choice as high-quality histomorphometry has demonstrated that many spacings in the CC are even smaller that 24 μm^46^. However, we felt the safest strategy was to employ the algorithm by Chu et al.^44^ that has been validated in the assessment of the CC flow signal.

The final binarized image was thus imported in Fiji ImageJ software version 2.0.0 (National Institutes of Health, Bethesda, MD; available at http://rsb.info.nih.gov/ij/index.html) and processed with the ‘Analyze Particles’ command, in order to count and measure the flow signal voids in a circular region of interest centered on the fovea (diameter of 5.0 mm). The percentage of flow deficits (FD%) and the average size of the flow deficits were calculated. As also the CC assessment may be significantly affected in regions with drusen because of shadowing artifacts, in the central 5 mm diameter of intermediate AMD eyes, the CC measurements were also separately performed in the “drusen-free” region (Figs. 1, 2, 3).

### Statistical analysis

All quantitative variables were reported as mean and standard deviation (SD) and/or median and interquartile range (IQR) in the Results section and in Table 1.

**Table 1:** Tested variables in controls and age-related macular degeneration patients.

	Controls	Intermediate AMD	
		Whole region	Drusen-free region
EZ normalized reflectivity	0.85 ± 0.08 0.86 (0.79–0.92)	0.76 ± 0.10 0.76 (0.71–0.84)	0.77 ± 0.10 0.76 (0.71–0.84)
		< .0001^a^	< .0001^a^
Choriocapillaris FD%	19.2 ± 6.2 20.7 (13.9–23.5)	24.1 ± 5.3 23.9 (21.9–28.1)	24.0 ± 5.4 23.9 (21.9–28.1)
		0.001^a^	0.001^a^
Choriocapillaris FD average size (μm^2^)	612.0 ± 72.6612.0 ± 72.6 590.4 (576.4–639.6)	898.2 ± 289.6898.2 ± 289.6 822.2 (690.8–943.4)	747.0 ± 151.0 748.6 (639.6–832.4)
		< .0001^b^	< .0001^b^

Data are presented as mean ± SD (standard deviation) and median (interquartile range).

AMD: age-related macular degeneration; EZ: ellipsoid zone; FD%: percentage of flow deficits.

^a^Independent samples T test—comparison with controls.

^b^Independent samples Mann–Whitney U test—comparison with controls.

To detect deviations from a normal distribution, Shapiro–Wilk’s test was performed for all variables. Independent samples T test and non-parametric Mann Whitney U test were conducted to investigate differences in continuous variables between the AMD and control groups.

Spearman’s correlation coefficient was used to assess correlations between EZ normalized reflectivity and other variables.

Statistical calculations were performed using Statistical Package for Social Sciences (version 20.0, SPSS Inc., Chicago, IL, USA). The chosen level of statistical significance was p < 0.05.

The sample size of the study was tested to be proper for a mean difference between groups of almost 10%, a power of 80% and type I error rate (α) of 5%.

## Results

### Characteristics of patients included in the analysis

Of the 70 patients (70 eyes) included in this analysis, 35 (21 female) were diagnosed with intermediate AMD and 35 (22 female) were healthy controls. In the intermediate AMD group, 27 patients presented with bilateral intermediate AMD, whereas 8 patients presented with neovascular AMD in the fellow eye. Mean ± SD age was 74.1 ± 6.8 years [range 64–88 years] in the intermediate AMD group and 72.1 ± 6.0 years [range 62–85 years] in the control group (p = 0.206).

In intermediate AMD eyes, the drusen area was 0.72 ± 0.73 mm^2^ [median = 0.44 mm^2^; IQR = 0.1–1.34 mm^2^], while the drusen volume was 0.026 ± 0.028 mm^3^ [median = 0.012 mm^3^; IQR = 0.003–0.049 mm^3^], as evaluated using the advanced RPE analysis software. The percent of area occupied by drusen in the central 5 mm diameter was 3.8 ± 3.2%.

### Comparison between the two groups

The normalized EZ reflectivity was 0.76 ± 0.10 [median = 0.76; IQR = 0.71–0.84] in the intermediate AMD group and 0.85 ± 0.08 [median = 0.86; IQR = 0.79–0.92] in the control group (p < 0.0001). As specified above, assuming that the assessment of the EZ reflectivity and CC flow signal may be challenging in the “drusen” region, we performed a separate analysis considering only the “drusen-free” region. In this analysis, the normalized EZ reflectivity was 0.77 ± 0.10 [median = 0.76; IQR = 0.71–0.84] in the “drusen-free” region (p < 0.0001 vs healthy controls).

The CC FD% was significantly increased in intermediate AMD eyes compared to control eyes (24.1 ± 5.3%, median = 23.9% and IQR 21.9–28.1% in the intermediate AMD group; 19.2 ± 6.2%, median = 20.7% and IQR 13.9–23.5% in the control group; p = 0.001). Similar differences were detected in the ”drusen-free” region (Table 1).

The average size of the CC flow signal deficits was increased in intermediate AMD eyes (898.2 ± 289.6 μm^2^, median: 822.2 μm^2^ and IQR: 690.8–943.4 μm^2^ in the intermediate AMD group; 612.0 ± 72.6 μm^2^, median: 590.4 μm^2^ and IQR: 576.4–639.6 μm^2^ in the normal group; p < 0.0001). The average FD size was significantly increased also considering the region without drusen (747.0 ± 151.0 μm^2^, median: 748.6 μm^2^ and IQR: 639.6–832.4 μm^2^), as compared with control eyes (p < 0.0001) (Table 1). The CC FD% and EZ normalized reflectivity were 26.5 ± 6.8% and 0.71 ± 0.11 in the regions with drusen, respectively.

### Correlation analysis

Considering the whole 5-mm-diameter region of interest (ROI), the EZ normalized reflectivity was inversely correlated with the drusen area (ρ = − 0.371 and p = 0.027) and volume (ρ = − 0.388 and p = 0.021), while it was not associated with both the CC FD percentage (ρ = 0.257 and p = 0.102) and average size (ρ = − 0.088 and p = 0.715). In the analysis considering only the “drusen-free” region, the EZ normalized reflectivity was inversely correlated with the CC FD% (ρ = − 0.340 and p = 0.020), but was not correlated with average FD size (ρ = 0.050 and p = 0.765).

In control eyes, the EZ normalized reflectivity was not associated with the CC variables (ρ = − 0.075 and p = 0.668 for FD% and ρ = 0.007 and p = 0.969 for FD average size).

## Discussion

In this retrospective case–control study we investigated the EZ reflectivity in intermediate AMD and healthy eyes. Our results showed that photoreceptors are affected in intermediate AMD eyes. Importantly, the EZ reflectivity was also reduced in areas without drusen, which may suggest that these eyes experience an early photoreceptor alteration impacting reflectivity. Furthermore, we also demonstrated a moderate relationship between CC flow signal and EZ normalized reflectivity in the “drusen-free” region of intermediate AMD eyes, whereas control eyes did not display a similar association. Hence, our findings may imply a pathological and early connection between CC and photoreceptors in these eyes.

Data from a number of studies using distinct approaches indicate that photoreceptors may be affected in AMD eyes^15,47^. Histopathological studies have demonstrated that photoreceptor alterations increase in presence of AMD. Curcio and colleagues^15^ have extensively studied the quantity of photoreceptors in eyes with mid- to late-stage AMD. They analyzed postmortem eyes from 7 donors (13 eyes) with AMD, including 5 non-exudative AMD eyes, and their findings were compared with age-matched controls. They observed that eyes with drusen were characterized by a significant reduction in photoreceptor number and that this decrease was mainly confined to the parafovea^15^. Importantly, there was heterogenicity in photoreceptor loss among donors, as in two donors, rod loss exceeded cone loss at most parafoveal locations, and in one donor, rod density was normal and cone density was reduced^15^. Using adaptive optics scanning laser ophthalmoscope, Boretsky et al.^47^ investigated 4 patients with AMD (one of these patients was affected by intermediate AMD). In the latter study, the authors demonstrated a progressive reduction in photoreceptors’ density throughout the successive AMD stages^47^. Furthermore, using structural OCT, Rogala and colleagues^48^ illustrated an extensive outer retinal thinning in intermediate AMD eyes, as compared with age-matched controls. Finally, a functional impairment in both rod- and cone- mediated vision has been extensively demonstrated in early/intermediate AMD eyes^49–51^.

We add to the literature by reporting a analysis of the photoreceptor damage in intermediate AMD eyes. Importantly, we quantified photoreceptor alteration by separately investigating regions with and without drusen. In order to provide an objective quantification of this damage, we measured the normalized reflectivity of the *en face* structural OCT image segmented at the level of the EZ. This assessment is not without challenges, including the presence of several factors that might impact on the OCT brightness and confound comparisons among subjects. This obstacle was resolved by “normalizing” the images—a technique that has been successfully employed in several prior studies, including in intermediate AMD eyes^16,29,34–36^.

Assessment of the EZ reflectivity has been used to investigate photoreceptor structure in different disorders, including macular telangiectasia (MacTel) type 2^52^, macular hole^53,54^, and AMD^16,28,55^. Pappuru et al.^28^ demonstrated that a lower EZ reflectivity is associated with worse visual acuities in eyes with intermediate AMD. In addition, the EZ reflectivity was demonstrated to be positively associated with ganglion cell loss, this supporting the postreceptoral hypothesis as rationale for the inner retinal impairment occurring in intermediate AMD eyes^16^. Noteworthy, there is uncertain on the presence of ganglion cell loss in early/intermediate AMD eyes, as previous histologic findings suggest a loss in ganglion cells only in long-standing advanced AMD^56^, Furthermore, one previous study reported on the correlation between EZ integrity and retinal sensitivity in AMD eyes^57^. The authors of the latter study proved that retinal sensitivity significantly correlated with the status of the EZ in both early and late stages of AMD. Thus, the functional significance of the EZ in AMD eyes would seem to be adequately established.

We confirmed that photoreceptors are affected in intermediate AMD eyes. Importantly, we provided a topographical analysis of the normalized EZ reflectivity. Importantly, when we compared the normalized reflectivity of drusen-free areas to control eyes, it was significantly reduced, indicating that the photoreceptors localized in the regions without drusen also are abnormal in AMD patients. These results seem to provide imaging evidence to support the observations that photoreceptors are extensively affected in intermediate AMD eyes.

Although OCTA has significantly improved our capability to investigate the CC, many challenges must be addressed in analyzing the CC vasculature. In order to quantify the CC flow signal, *en face* OCTA images of this vascular layer may be binarized to separate pixels constituting these images into two groups: one that represents regions of flow signal and another representing signal deficits. Although a global threshold may be used to obtain binarized images of the CC, this approach may be problematic in presence of drusen that may cause local changes in image brightness that may be erroneously identified as FDs. In order to mitigate this issue, we employed an algorithm to compensate for the signal attenuation in the CC and we adopted a local threshold (Phansalkar method) which uses a small window rather than the whole image to determine the threshold for binarization. A study by Chu and colleagues^44^ has recently assessed the proper use of this threshold for CC binarization. In details, they demonstrated that applying a window radius of 2–4 pixels is the best strategy for analyzing 6 × 6-mm scans with a 1024 × 1024 size. Assuming this, we applied a window radius of 3 pixels (~ 103 μm^2^).

In recent years, OCTA technology has granted a considerable resource to study AMD. Several previous OCTA studies have described CC alterations in early and intermediate AMD eyes^20–23,43,58,59^. In agreement with these studies, we confirmed that intermediate AMD eyes are characterized by CC impairment.

One of the most notable observations from our study was that the reduction in normalized EZ reflectivity is associated with CC flow signal in the “drusen-free” areas. As stated above, we chose to perform a separate analysis in the regions without drusen in order to investigate the relationship between CC flow signal and photoreceptors after restricting confounding factors (see the “Introduction” section for further details). Of note, our analysis was not able to incorporate the function of the outer blood-retina barrier, as we provided a rough correlation between the CC flow signal and photoreceptor structure. The relationship between these two structures was displayed in intermediate AMD eyes, while the absence of this association in healthy subjects may recommend the presence of a pathological dysregulation in the AMD group. These findings provided further evidence that photoreceptors and choriocapillaris are elements of a symbiotic unit in which dysfunctions of their components are strictly correlated.

Our study has limitations including the absence of a longitudinal evaluation. Another limitation of our image analysis is that media opacities and other causes of signal attenuation may have artifactiously caused a simultaneous reduced EZ reflectivity and CC flow signal, this producing a false positive correlation between these two structures. However, our algorithms were aimed at reducing this effect, as follows: (i) angiographic CC images were compensated for the signal attenuation using structural information, and (ii) the normalized EZ reflectivity was obtained with a process which compensates for the signal attenuation by using the RNFL and vitreous reflectivity. Furthermore, *en face* structural OCT and OCTA images must be interpreted with caution owing to a variety of image artifacts in the presence of drusen^60^. Thus, we felt the safer strategy was to perform an additional analysis limited to the “drusen-free” region. However, it must be noted that also drusen-free regions may be characterized by subtle changes as histopathology demonstrated the presence of basal linear deposits in areas without drusen^61^. Furthermore, the quantification of EZ reflectivity must be interpreted with caution as the OCT beam directionality may significantly influence this assessment^62^. Future studies will clarify if this aspect may have influenced our findings. Finally, we failed to provide a sophisticated topographical analysis as we only separately analyzed the regions without drusen. However, this is the first study to use imaging to directly correlate CC flow signal with photoreceptor damage in intermediate AMD eyes. We believe that the findings in our paper will be of help in further hypothesis generation and future study design.

In summary, in this SS-OCT structural and angiography study of the photoreceptors and CC, we observed that eyes with intermediate AMD have a diffuse reduced normalized EZ reflectivity. Importantly, the reduction in CC flow signal seems to be strongly associated with photoreceptor damage in areas without drusen. Future studies with longitudinal follow up may provide further insight into the interrelationship between photoreceptors, the choriocapillaris, and the pathogenesis of AMD.

## Data Availability

The data used to support the findings of this study are available from the corresponding author upon request.
